# Risk Factors for Fracture in Patients with Coexisting Chronic Kidney Disease and Type 2 Diabetes: An Observational Analysis from the CREDENCE Trial

**DOI:** 10.1155/2022/9998891

**Published:** 2022-05-27

**Authors:** Tamara K. Young, Nigel D. Toussaint, Gian Luca Di Tanna, Clare Arnott, Carinna Hockham, Amy Kang, Aletta E. Schutte, Vlado Perkovic, Kenneth W. Mahaffey, Rajiv Agarwal, George L. Bakris, David M. Charytan, Hiddo J. L. Heerspink, Adeera Levin, Carol Pollock, David C. Wheeler, Hong Zhang, Meg J. Jardine

**Affiliations:** ^1^The George Institute for Global Health, UNSW, Sydney, Australia; ^2^Department of Nephrology, The Royal Melbourne Hospital, Parkville, Victoria, Australia; ^3^Department of Medicine (RMH), University of Melbourne, Parkville, Victoria, Australia; ^4^Department of Cardiology Royal Prince Alfred Hospital, Sydney, Australia; ^5^The George Institute for Global Health, Imperial College London, UK; ^6^Prince of Wales Hospital, Randwick, Australia; ^7^Faculty of Medicine, UNSW, Australia; ^8^Stanford Center for Clinical Research, Department of Medicine, Stanford University School of Medicine, Stanford, CA, USA; ^9^Indiana University School of Medicine and Veterans Affairs Medical Center, Indianapolis, Indiana, USA; ^10^Department of Medicine, University of Chicago Medicine, Chicago, Illinois, USA; ^11^Nephrology Division, NYU School of Medicine and NYU Langone Medical Center, New York, New York, USA; ^12^University of Groningen, Groningen, Netherlands; ^13^Division of Nephrology, University of British Columbia, Vancouver, British Columbia, Canada; ^14^Kolling Institute of Medical Research, Sydney Medical School, University of Sydney, Australia; ^15^Royal North Shore Hospital, St Leonards, New South Wales, Australia; ^16^Department of Renal Medicine, UCL Medical School, London, UK; ^17^Renal Division of Peking University First Hospital, Beijing, China; ^18^NHMRC Clinical Trial Centre, University of Sydney NSW, Australia; ^19^Concord Repatriation General Hospital, Sydney, Australia

## Abstract

**Background:**

The fracture pathophysiology associated with type 2 diabetes and chronic kidney disease (CKD) is incompletely understood. We examined individual fracture predictors and prediction sets based on different pathophysiological hypotheses, testing whether any of the sets improved prediction beyond that based on traditional osteoporotic risk factors.

**Methods:**

Within the CREDENCE cohort with adjudicated fracture outcomes, we assessed the association of individual factors with fracture using Cox regression models. We used the Akaike information criteria (AIC) and Schwartz Bayes Criterion (SBC) to assess six separate variable sets based on hypothesized associations with fracture, namely, traditional osteoporosis, exploratory general population findings, cardiovascular risk, CKD-mineral and bone disorder, diabetic osteodystrophy, and an all-inclusive set containing all variables.

**Results:**

Fracture occurred in 135 (3.1%) participants over a median 2.35 [1.88–2.93] years. Independent fracture predictors were older age (hazard ratio [HR] 1.04, confidence interval [CI] 1.01–1.06), female sex (HR 2.49, CI 1.70–3.65), previous fracture (HR 2.30, CI 1.58–3.34), Asian race (HR 1.74, CI 1.09–2.78), vitamin D therapy requirement (HR 2.05, CI 1.31–3.21), HbA1c (HR 1.14, CI 1.00–1.32), prior cardiovascular event (HR 1.60, CI 1.10–2.33), and serum albumin (HR 0.41, CI 0.23–0.74) (lower albumin associated with greater risk). The goodness of fit of the various hypothesis sets was similar (AIC range 1870.92–1849.51, SBC range 1875.60–1948.04).

**Conclusion:**

Independent predictors of fracture were identified in the CREDENCE participants with type 2 diabetes and CKD. Fracture prediction was not improved by models built on alternative pathophysiology hypotheses compared with traditional osteoporosis predictors.

## 1. Introduction

Minimal trauma fractures are a global health issue, with an annual toll of 9 million fractures, 740,000 associated-deaths, and accounting for a loss of 1.75 million disability-adjusted life-years [1]. The concept of osteoporosis, a systemic skeletal syndrome incorporating low bone mass, microarchitectural distortion of bone tissue, and increased susceptibility to fracture, encapsulates the understanding of pathophysiological factors leading to minimal trauma fractures in the general population [1–4]. Established risk factors for minimal trauma fracture in the general population include female sex, advanced age, some classes of medication, and a previous history of fracture [3–5]. Other more exploratory clinical associations include alterations in circulating biomarkers, including surrogate markers of frailty and compromised nutrition reflected by a low serum albumin concentration [6].

Fracture rates in people with chronic kidney disease (CKD) or type 2 diabetes mellitus are up to fourfold higher compared with the general population [7–10]. The higher rates are consistent with hypotheses that the pathophysiology of poor bone quality and disrupted bone architecture is distinct in CKD and type 2 diabetes as this patient group has a further elevated risk compared to the general population [9, 11–14]. The concept of chronic kidney disease–mineral and bone disorder (CKD-MBD) incorporates the diverse pathologies associated with CKD, including markers of impaired bone mineralisation and altered states of bone turnover [14]. There is, however, a wide spectrum in both alterations of bone microarchitecture and biochemical abnormalities observed in patients with CKD-MBD resulting in inherent difficulties in fracture risk prediction [15]. Concepts of a diabetic osteodystrophy are not as well formulated as for CKD-MBD although recent interest has focussed on a potential direct role of microvascular disease on bone health [16]. In this hypothesis, alterations to bone microarchitecture occur in people with diabetes via a plausible mechanism of compromised vasculature, as also occurs with established mechanisms of diabetic macrovascular disease. Markers of macrovascular risk may also signify increased risk for microvascular disease although the association of these markers with fracture has not been examined in large cohorts.

Microvascular disease could conceivably contribute to poor bone health in CKD as well as diabetes. Like type 2 diabetes, CKD is also associated with vascular complications, with rates of cardiovascular complications increasing roughly 2.5-fold for every halving of estimated glomerular filtration rate (eGFR) [17]. The vascular associations of CKD are better described for macrovascular disease than microvascular disease, but a potential microvascular pathological process within bone is plausible [18]. Derangement in metabolic parameters of mineral metabolism associated with CKD may pose a risk of vessel calcification as well as for poor bone mineralisation [14, 19].

SGLT-2 inhibitors are a novel class of medication that induce glycosuria by blocking glucose resorption in the proximal tubule, subsequently inducing changes to parameters of bone mineral metabolism such as phosphate clearance, fibroblast growth factor 23 (FGF23), and parathyroid hormone levels [20, 21]. A previous study in older patients with diabetes has demonstrated that canagliflozin was associated with a small but statistically decrease in total hip bone mineral density, over a 104-week period [22]. Despite this recognised disturbance to clinical parameters, 2 previous meta-analyses have not found an overall increased risk for this class of medication and the occurrence of fracture [23, 24]. There was an association in one of the two trials in the CANVAS program that also utilised canagliflozin, although there was significant heterogeneity in the effects on fracture (*p* = 0.005) between CANVAS (*n* = 4330: HR 1.55 [95% CI 1.21, 1.97]) and CANVAS-R (*n* = 5812: HR 0.86 [95% CI 0.62, 1.19]), and the reasons for the association in the CANVAS trial were not clear, possibly representing a chance finding [25, 26].

The CREDENCE trial assessed the impact of a sodium-glucose cotransporter-2 (SGLT2) inhibitor, canagliflozin, on cardiovascular and kidney outcomes in participants with known CKD and type 2 diabetes. In this trial, canagliflozin treatment was not associated with an increased risk of fracture (hazard ratio [HR] 0.98, confidence intervals [CI] 0.70-1.37) [27]. The CREDENCE participants represent a large cohort with both established type 2 diabetes and CKD with comprehensive follow-up. Fractures were a prespecified, reportable, and adjudicated event in the CREDENCE trial, making this cohort ideal for examining the relative association of markers of poor bone health.

Traditional risk factors for fracture in the general population may not apply to this patient group, and so we sought indirect evidence on whether other processes might be linked to fracture risk. In this post hoc analysis, we sought to explore the aetiology of fracture pathophysiology in people with type 2 diabetes and CKD. We aimed to test whether grouping risk factors according to aetiological hypothesis sets might improve the ability to predict fracture in this cohort: the aetiological hypothesis sets tested were composed of specific factors associated with the disease-specific processes of diabetes, CKD-MBD or vascular disease, or exploratory factors observed in the general population.

## 2. Material and Methods

### 2.1. Study Overview and Design

The CREDENCE trial was a multicentre, randomised, double-blinded controlled trial designed to assess the safety and efficacy of canagliflozin in participants with type 2 diabetes and known CKD (ClinicalTrials.gov NCT02065791) [27].

### 2.2. Study Population

A total of 4401 individuals underwent randomisation at 690 sites in 34 countries between March 2014 and May 2017. The trial recruited participants with established type 2 diabetes of age greater than 30 years old with no upper age limit. All participants had underlying CKD, defined by the entry criteria of an eGFR between 30 and 90 mL/min/1.73m^2^ and macroalbuminuria (urinary albumin-to-creatinine ratio > 300 to 5000 mg/g).

### 2.3. Randomisation and Conduct of the CREDENCE Trial

Participants were randomised to canagliflozin 100 mg or matching placebo, and study treatment was continued until commencement of dialysis, receipt of a kidney transplant, occurrence of diabetic ketoacidosis, pregnancy, or receipt of disallowed therapy or study conclusion. Follow-up occurred at 3, 13, and 26 weeks, then every 13 weeks. Glycaemic management was at the discretion of the treating physician and in line with applicable local guidelines. The use of other therapies was according to best practices followed throughout the course of the study and instituted according to local guidelines and policies. Local institutional ethics committees approved the trial protocols at each site. All participants provided written informed consent. The trial was conducted according to the principles outlined in the Declaration of Helsinki.

### 2.4. Main Trial Outcomes

The primary outcome of the CREDENCE trial was the composite of kidney failure, doubling of serum creatinine or death from kidney or cardiovascular causes, with additional cardiovascular and kidney events as secondary outcomes.

### 2.5. Study Outcome for This Analysis

The outcome for these analyses was the occurrence of an initial fracture during the study period. Fracture was prespecified as a key safety outcome and serious adverse event in the CREDENCE trial and coded using the Medical Dictionary for Regulatory Activities (MedDRA), from randomisation until 30 days after the last date of blinded study medication. Information collected included the site and type of fracture. Events were independently adjudicated by a committee whose members were unaware of trial group assignments. This consisted of two independent reviewers, who confirmed the event occurrence and region, with a tie breaker system if the reviewers disagreed on the outcome.

### 2.6. Statistical Analysis

A time to event analyses for the occurrence of the first fracture during trial follow-up was performed. All participants who received at least one dose of canagliflozin or placebo through to the end of the trial (i.e., modified intention-to-treat population) were included.

Fracture was analysed using a stratified Cox proportional hazards regression model, with stratification according to eGFR category at screening [1] ≥30 to <45 mL/min/1.73m^2^ [2], 45 to <60 mL/min/1.73m^2^, or [3] 60 to <90 mL/min/1.73m^2^, in accordance with previous safety analyses. Estimated glomerular filtration (eGFR) was calculated using the CKD-EPI (CKD Epidemiology Collaboration) formula. Hazard ratios (HRs) and 95% confidence intervals (CIs) were estimated for participants assigned to canagliflozin versus participants assigned to placebo, and annualised incidence rates were calculated per 1000 patient-years. Proportional hazard assumptions were checked by visual assessment of the log cumulative-hazard functions and by Kolmogorov-type supremum test.

Prior to considering all risk factors for fracture in this trial population, the effect of canagliflozin versus placebo according to multiple different baseline subgroups was assessed by fitting Cox-proportional hazard models in each of the prespecified subgroups, then fitting an interaction term to the model to assess for heterogeneity. Subgroups were selected according to plausible associations with the aetiological hypothesis, including traditional and exploratory risk factors.

We avoided any use of model building based on automatic procedures. Candidate variables for all modelling were selected because of their association with the aetiological hypotheses, namely:

- Traditional, well-established fracture risk factors of age, sex, and self-reported history of any fracture
- Exploratory general population factors of race, serum albumin, and various medication use (proton pump inhibitors [PPI], thyroid hormone replacement, calcium supplement, vitamin D therapy [defined by any form of vitamin D treatment, supplementation or analogue use], calcium supplement use, and beta blockers)
- Associations of CKD-MBD including eGFR, urine albumin-to-creatinine ratio, and baseline biochemical parameters associated with changes in bone and mineral metabolism (alkaline phosphatase [ALP], serum calcium, magnesium, phosphate, bicarbonate, sodium, and urate)
- Diabetic-osteodystrophy-related factors including disease duration, baseline glycated haemoglobin (HbA1c), baseline body mass index (BMI), retinopathy, neuropathy, and insulin use
- Established clinical associations of cardiovascular disease including baseline systolic blood pressure, low-density lipoprotein (LDL)-cholesterol, triglycerides, history of smoking, cholesterol-lowering statin use, and underlying history of cardiovascular disease

Variables exhibiting collinearity on inspection of a scatter plot for linear variables were excluded. These included total cholesterol, high-density lipoprotein- (HDL-) cholesterol, diastolic blood pressure, and geographic region, which were excluded in favour of low-density lipoprotein- (LDL-) cholesterol, systolic blood pressure, and race.

Only a small number of participants were taking bisphosphonates at baseline (*n* = 19), and as this violated the supremum proportional hazards assumption, this variable was not included in the models. No participants were recorded as receiving other antiresorptives such as denosumab or osteoanabolics such as teriparatide, at baseline.

Univariable models were used to assess the associations of candidate risk factors with fracture. Multivariable models were created that firstly tested the traditional osteoporotic set as the base-case and then tested each of the variable sets described above in combination with the traditional osteoporotic set, as well as an overall set of all tested variables.

The six models were compared by examining the Akaike information criteria (AIC) and Schwartz Bayes Criterion (SBC). All models were adjusted for treatment group allocation. Complete case data was analysed, and where there was data missing for candidate variables, these cases were omitted. All analyses were performed with the use of SAS software, version 7.1 (SAS Institute). A *p* value of less than 0.05 was deemed statistically significant for all analyses.

## 3. Results

### 3.1. Population and Fracture Incidence

Of the 4401 participants randomised in the CREDENCE trial, 4397 received at least one dose of medication and were included in this analysis as the on-study population. The mean age was 63 ± 9 years, and 33.9% of the patients were women (Supplementary Table 1) including 1201 women aged over 55. The mean HbA1c value was 8.3%. The mean eGFR was 56.2 mL/min/1.73 m^2^, while there were 1311 participants with an eGFR < 45 mL/min/1.73m^2^. The cohorts were followed for a median of 2.35 years (interquartile range [IQR] 1.88 to 2.93 years).

Overall, a total of 159 fractures occurred in 135 (3.1%) participants. The event rate for initial fracture was 11.9 fractures per 1000 patient years (Supplementary Figure 1). The mean time to initial fracture was 483.6 ± 320.7 days. The most common initial fracture site was the lower limb, comprising around half of the events (64/135, 47.4%), followed by the upper limb (39/135, 28.9%). Only 9 participants experienced a spinal fracture (6.7%) (Supplementary Table 2).

There was no evidence of an effect of canagliflozin on fracture, with 67 participants randomised to canagliflozin and 68 randomised to placebo experiencing this outcome (HR 0.98 (CI 0.70–1.37) (Supplementary Figure 1). The lack of an effect of canagliflozin on fracture risk was generally consistent across all studied subgroups, including subgroups defined by the traditional fracture risk factors of age and previous history of fracture (Supplementary Figure 2).

### 3.2. Risk Factors for Fracture

#### 3.2.1. Univariable Modelling

In univariable regression models, traditional variables associated with fracture included age (HR 1.03, CI 1.01–1.05, *p* < 0.001), female sex (HR 2.3, CI 1.64–3.23, *p* < 0.001), and a history of previous fractures (HR 2.31, CI 1.60–3.33, *p* < 0.001). Exploratory general population factors associated with fracture included lower serum albumin (HR 0.49, CI 0.32–0.74, *p* < 0.001) and vitamin D therapy (HR 2.1, CI 1.39–3.19, *p* < 0.001). None of the CKD-MBD factors were associated with fracture. In particular, there was no association with lower eGFR (*p* = 0.09) or with baseline urine albumin-to-creatinine ratio (*p* = 0.882). Among the diabetic osteodystrophy-related factors, a higher HbA1c was associated with an increased fracture risk (HR 1.13, CI 1.00–1.28, *p* = 0.05) but no other diabetes-related parameters were statistically significant. Among risk factors for cardiovascular disease, only a history of cardiovascular disease was associated with fracture (HR 1.51, CI 1.07–2.14, *p* = 0.002) (Supplementary Table 3).

#### 3.2.2. Multivariable Modelling of Variable Sets

The multivariable model of traditional osteoporotic risk factors demonstrated all factors were independently predictive of fracture (Table 1(a)).

The multivariable model that included exploratory risk factors in addition to the base-case found that all traditional risk factors and some general exploratory factors—Asian race (HR 1.72, CI 1.13–2.62, *p* = 0.012), lower serum albumin (HR 0.47, CI 0.30–0.72, *p* < 0.001), and vitamin D therapy (HR 1.96, CI 1.27–3.03, *p* = 0.0002)—were significantly associated with fracture (Table 1(b)).

The inclusion of CKD-MBD factors in addition to traditional osteoporosis risk factors did not yield any significant associations with biochemical or CKD-related parameters after accounting for the traditional factors (Table 1(c)). Similarly, none of the diabetic-osteodystrophy-related factors were significantly associated with fracture after accounting for the traditional osteoporosis factors (Table 1(d)).

A history of cardiovascular disease was associated with fracture after accounting for the traditional osteoporosis risk factors (HR 1.45, CI 1.02–2.07, *p* = 0.04) but none of the cardiovascular risk factors, including smoking history, baseline lipid levels, or systolic blood pressure, were significantly associated with fracture (Table 1(e)).

**Figure 1:**
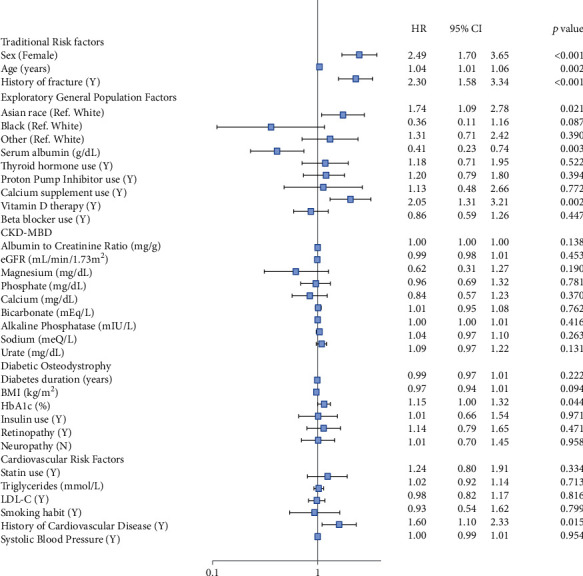
Multivariable Cox-proportional model for all variables and fracture. CKD-MBD: chronic kidney disease-mineral bone disorder; CI: confidence interval; eGFR: estimated glomerular filtration rate (note: estimated glomerular filtration was calculated using the CKD-EPI (CKD Epidemiology Collaboration) formula); HR: hazard ratio; LDL-C: low-density lipoprotein-cholesterol.

In the overall, all-inclusive model, results of significance testing were largely consistent with those seen in separate variable set testing. The traditional osteoporosis risk factors remained significantly associated with fracture with HRs that approximated the base model of the traditional osteoporosis risk factors alone (Figure 1).

The assessments of fit of the different models did not vary greatly. The model with the best numerical AIC fit was the overall model which included all factors (1849.51). The CKD-MBD model had the weakest numerical fit (1870.92) while the base-case (traditional osteoporotic factors alone) had an intermediate fit (1863.98) although the difference across the range of best to worst fit was marginal (Table 2).

## 4. Discussion

Systematic identification and adjudication of fracture events in the CREDENCE trial allowed a rigorous evaluation of the rates and associations of fracture in a high-risk multicentre population with type 2 diabetes, macroalbuminuria, and an eGFR range between 30 and 90 mL/min/1.73m^2^. Canagliflozin had no impact on fracture rates in the overall trial population or across a broad range of subgroups. In this clinically well-characterised population with both type 2 diabetes and CKD, we confirmed that fracture was independently predicted by the traditional osteoporotic risk factors of age, female sex, and prior history of fracture, whether tested alone or in combination with other potential risk factors. Additional variables that were independently associated with fracture risk after accounting for traditional osteoporotic factors included Asian race, lower serum albumin, use of a vitamin D therapy, HbA1c, and a history of cardiovascular disease. In all models, while a number of less established risk factors were associated with fracture and may indicate the presence of novel pathophysiological pathways, most or all of the ability to predict fracture risk in this cohort was determined by well-established, traditional predictors of fracture.

The fracture rate of 3.1% in this trial of participants with diabetic kidney disease was roughly twice as high as that seen in other trials of SGLT2 inhibitors which were conducted in people with high-risk cardiovascular disease and underlying type 2 diabetes, and similar to that seen in the DAPA-CKD trial that also recruited participants with known CKD [24, 28]. Fracture rates are increased in people with reduced kidney function, defined as an eGFR less than 60 mL/min/1.73m^2^ [8, 29]. A recent meta-analysis also reported an increased risk of fracture as kidney function deteriorates, with highest rates in patients with stage 5 CKD [30]. As eGFR declines, biochemical indicators of altered bone mineralisation, such as impaired phosphate regulation and secondary hyperparathyroidism, are increasingly common, posing an increased risk of poor bone quality. Despite this, neither eGFR nor albuminuria were independent predictors for fracture in the CREDENCE cohort, after accounting for the traditional osteoporotic risk factors. Furthermore, we did not find associations with baseline serum urate, bicarbonate, sodium, alkaline phosphatase, calcium, or phosphate levels, all of which have been previously implicated in altering bone mineralisation or increased fracture risk [19]. It remains possible that potential factors associated with CKD-MBD may predict fracture in populations with more advanced CKD.

This analysis provides further information regarding the safety of canagliflozin, with no observed increase in fracture following exposure compared to those receiving placebo, over a mean of 2-year follow-up. This is concordant with a recent meta-analysis of SGLT2 inhibitors that did not show an overall risk of fracture [23]. Whilst a finding from one trial of the CANVAS program (CANVAS) did demonstrate an increased risk of fracture, despite extensive analysis of trial heterogeneity and participant characteristics, no clear explanation was found, and this may represent a chance finding [25]. Our findings have not provided any further insight into this observed outcome in the CANVAS program or provided any evidence for a canagliflozin-specific effect (within the class of SGLT2 inhibitors) on risk of fracture.

Newer hypotheses on fracture pathophysiology include pathways mediated by diabetes, CKD, and vascular disease (all conditions which were well represented in the CREDENCE cohort). Despite this, most predictors for fracture in this trial population are consistent with traditional risk factors for osteoporosis, including older age, female sex, and prior history of fracture [31]. In this analysis, some less-established factors also proved to be independently predictive of fracture. Lower serum albumin was associated with fracture, an association reported previously in a cohort with underlying cardiovascular disease [6], and may represent compromised nutritional status or an increased inflammatory status [32]. The association between HbA1c and fracture risk found in the CREDENCE cohort confirms a previous report from over 20,000 participants aged over 65 with type 2 diabetes, supporting the hypothesis that impaired glycaemic control or other associations with diabetes may lead to poorer bone quality [33].

Compromised vasculature has been postulated as a mechanism for poor bone quality in patients with type 2 diabetes that leads to an increased fracture risk [16, 17]. Whilst macrovascular disease is most commonly described at coronary and cerebrovascular sites, and in peripheral vasculature, other potential sites of involvement such as bone are plausible. This hypothesis provides an explanation for the alterations to bone microarchitecture that occur in patients with type 2 diabetes, compromising bone quality and increasing fracture risk [11, 12]. A previous analysis of a large prospective cohort study of individuals with high cardiovascular risk noted an increase in hip fractures with subclinical cardiovascular disease [34]. Smoking, an established cardiovascular disease risk factor, is also considered a traditional risk factor for osteoporosis and is part of risk prediction algorithms such as FRAX [35], yet was not associated with fracture in the current analysis. Whether the associations of a history of cardiovascular disease, poor diabetes control, and indeed low serum albumin with fracture reflect concurrent vascular compromise in bone, or serve as indicators of an overall burden of disease and frailty in these individuals, remains unclear.

Vitamin D deficiency is an extensively studied, traditional risk factor for fracture, albeit with no clear target level for fracture prevention [36]. Supplemental vitamin D was not found to reduce fracture risk or improve bone mineral density in a recent meta-analysis [37]; however, vitamin D replacement continues to be recommended for high-risk, vitamin D deplete individuals in major guidelines [3, 38]. In this analysis, our definition of vitamin D therapy included both calcitriol and cholecalciferol, with the former potentially used for management of secondary hyperparathyroidism. Indication bias may explain the association of vitamin D therapy with fracture. Additionally, the clinical implications are that supplementation alone may not be sufficient to reduce fracture risk and that individuals who have previously been vitamin D deficient remain at ongoing risk of fracture despite vitamin D therapy.

This analysis has some relevant limitations. Short trial follow-up time may have limited the power for analysis of safety outcomes such as fracture, as sufficient time may not have elapsed for subjects to develop this complication. The trial population only included participants with stage 2 and 3 CKD and may not be generalisable to people with more severe kidney impairment, who are more likely to have clinical features of CKD-MBD such as secondary hyperparathyroidism, or to people with type 2 diabetes but no underlying kidney impairment. Therefore, the results apply to people with coexisting type 2 diabetes and CKD (specifically an eGFR 30-90 and macroalbuminuria) and cannot necessarily be generalised to other populations. The CREDENCE trial was designed to test the effect of canagliflozin on cardiorenal outcomes and not on fracture, although fracture was a reportable adverse event. This trial recruited a significantly higher proportion of male participants and therefore relatively underrepresents women, a group with increased fracture risk in most studies. Due to small sample size, we were unable to compare outcomes for different sites of fracture. Some potential associations with fracture could not be assessed, including some biochemical parameters disrupted by CKD, such as vitamin D circulating concentration level or associated dosing, parathyroid hormone, fibroblast growth factor 23 (FGF-23), and bone-specific alkaline phosphatase. Additionally, an association with fracture and the results of bone imaging or bone mineral density assessment was not explored in this analysis, but may be useful for future studies. Whilst an effect of canagliflozin on fracture cannot be excluded, all models were adjusted for treatment group inclusion to minimise this source of bias. Strengths of this analysis include the study population, comprising of a cohort derived from a large randomised controlled trial, performed to a high standard at multiple international sites. The outcome of fracture was adjudicated by an independent committee who was unaware of trial group allocation. We were able to incorporate multiple components into this analysis when considering risk factors, including past medical history, medication use and biochemical results at study baseline, supporting a robust and comprehensive analysis.

## 5. Conclusion

Traditional osteoporotic risk factors predict fracture in patients with coexisting type 2 diabetes and stage 2-3 CKD. Whilst some additional factors such as a history of cardiovascular disease and lower serum albumin levels are associated with fracture, incorporating them into models built on alternative pathophysiology hypotheses does not meaningfully improve the ability to predict fracture.

## Figures and Tables

**Table 1:** Multivariable Cox-proportional model for the association between: (a) established traditional risk factors and fracture; (b) general population exploratory risk factors and fracture; (c) CKD-MBD factors and fracture; (d) diabetic osteodystrophy-related factors and fracture; e) cardiovascular risk factors and fracture.

Variable	1a) Traditional risk factors			1b) Exploratory risk factors			1c) CKD-MBD			1d) Diabetic osteodystrophy			1e) Cardiovascular risk factors		
	Hazard ratio	95% confidence interval	*p* value	Hazard ratio	95% confidence interval	*p* value	Hazard ratio	95% confidence interval	*p* value	Hazard ratio	95% confidence interval	*p* value	Hazard ratio	95% confidence interval	*p* value
Sex (female)	2.32	1.65–3.25	<0.001	2.26	1.60–3.19	<0.001	2.35	1.63–3.38	<0.001	2.25	1.59–3.17	<0.001	2.45	1.73–3.48	<0.001
Age (years)	1.03	1.01–1.05	0.001	1.04	1.02–1.06	<0.001	1.03	1.01–1.06	0.001	1.04	1.02–1.06	0.001	1.03	1.01–1.05	0.009
History of fracture (Y)	2.29	1.59–3.29	<0.001	2.19	1.51–3.17	<0.001	2.25	1.56–3.24	<0.001	2.34	1.62–3.39	<0.001	2.25	1.56–3.24	<0.001
Asian race (ref. white)				1.72	1.13–2.62	0.012									
Black (ref. white)				0.35	0.11–1.11	0.074									
Other (ref. white)				1.19	0.66–2.15	0.571									
Serum albumin (g/dL)				0.47	0.30–0.72	0.001									
Thyroid hormone use (Y)				1.13	0.69–1.85	0.634									
Proton pump inhibitor use (Y)				1.27	0.86–1.88	0.229									
Calcium supplement use (Y)				1.06	0.46–2.46	0.897									
Vitamin D therapy (Y)				1.96	1.27–3.03	0.002									
Beta blocker use (Y)				0.95	0.66–1.35	0.764									
Urine albumin to creatinine ratio (mg/g)							1.00	1.00–1.00	0.863						
eGFR (mL/min/1.73 m^2^)							0.99	0.97–1.01	0.238						
Magnesium (mg/dL)							0.67	0.34–1.30	0.234						
Phosphate (mg/dL)							1.04	0.76–1.42	0.822						
Calcium (mg/dL)							0.72	0.52–1.00	0.051						
Bicarbonate (mEq/L)							1.01	0.95–1.07	0.767						
Alkaline phosphatase (IU/L)							1.00	1.00–1.01	0.195						
Sodium (mEQ/L)							1.02	0.97–1.09	0.434						
Urate (mg/dL)							1.07	0.96–1.19	0.246						
Diabetes duration (years)										0.99	0.97–1.01	0.437			
BMI (kg/m^2^)										0.98	0.95–1.01	0.134			
HbA1c (%)										1.12	0.98–1.27	0.095			
Insulin use (Y)										1.22	0.81–1.82	0.34			
Retinopathy (Y)										1.17	0.82–1.68	0.376			
Neuropathy (y)										1.07	0.75–1.51	0.723			
Statin use (Y)													1.33	0.87–2.04	0.182
Systolic blood pressure (mmHg)													1.00	0.99–1.01	0.91
Triglycerides (mmol/L)													1.00	0.89–1.11	0.956
LDL-C (mmol/L)													0.99	0.83–1.18	0.893
Smoking habit (Y)													0.93	0.54–1.61	0.799
History of cardiovascular disease (Y)													1.45	1.02–2.07	0.039

For the base-case model of traditional risk factors, no cases were excluded. For the overall multivariable model, 43 cases were omitted due to missing data. BMI: body mass index; CKD-MBD: chronic kidney disease-mineral bone disorder; eGFR: estimated glomerular filtration rate (note: estimated glomerular filtration was calculated using the CKD-EPI (CKD Epidemiology Collaboration) formula); LDL-C: low-density lipoprotein-cholesterol; SBP: systolic blood pressure; UACR: urine albumin-to-creatinine ratio.

**Table 2:** Comparison of model performance.

	Sets of potential predictors based on aetiological hypotheses											
	Traditional osteoporotic		Exploratory general population		CKD-MBD factors		Diabetic osteodystrophy related factors		Cardiovascular risk factors		Overall model	
	Without covariates	With covariates	Without covariates	With covariates	Without covariates	With covariates	Without covariates	With covariates	Without covariates	With covariates	Without covariates	With covariates
AIC	1908.18	1863.98	1908.05	1849.65	1907.73	1870.92	1907.71	1867.43	1892.32	1852.63	1891.47	1849.51
SBC	1908.18	1875.6	1908.05	1887.42	1907.73	1908.69	1907.71	1896.48	1892.32	1881.6	1891.47	1948.04

AIC: Akaike information criteria; SBC: Schwartz Bayes Criterion.

## Data Availability

Data from this study is available in the public domain via the Yale University Open Data Access Project (http://yoda.yale.edu/). This includes deidentified individual participant data, data definition specification, annotated case report form, protocol with amendments, and primary statistical analysis plan.

## Supplementary Materials

Supplementary 1 Supplementary Figure 1: Kaplan-Meier curve for event of fracture.

Supplementary 2 Supplementary Figure 2: fracture events according to canagliflozin versus placebo across baseline subgroups.

Supplementary 3 Supplementary Table 1: baseline characteristics of participants. Supplementary Table 2: fractures according to the skeletal site. Supplementary Table 3: univariate Cox-proportional models for fracture.
