# Supplementary figures and images for: Risk Factors for Fracture in Patients with Coexisting Chronic Kidney Disease and Type 2 Diabetes: An Observational Analysis from the CREDENCE Trial
Source: J Diabetes Res. 2022 May 27;2022:9998891. doi: 10.1155/2022/9998891 (PMC9168808; doi:10.1155/2022/9998891)

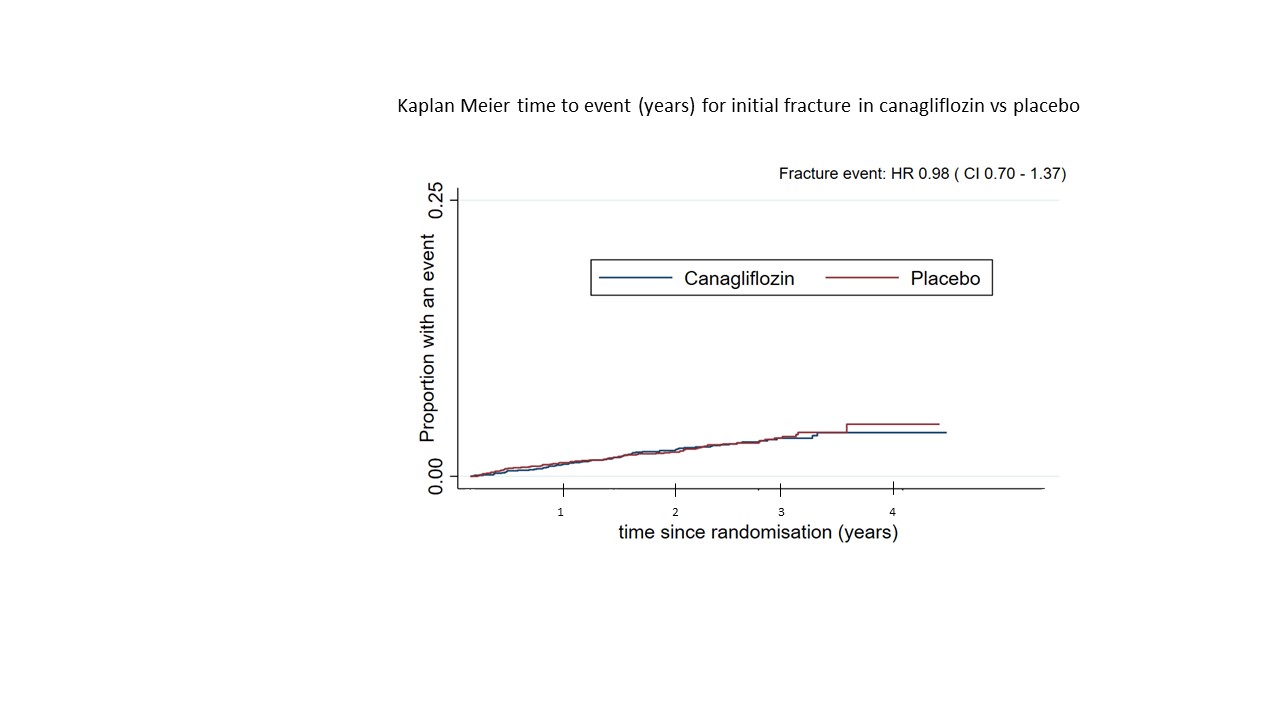

Supplement: Supplementary 1 — Supplementary Figure 1: Kaplan-Meier curve for event of fracture. [file 9998891.f1.jpg]

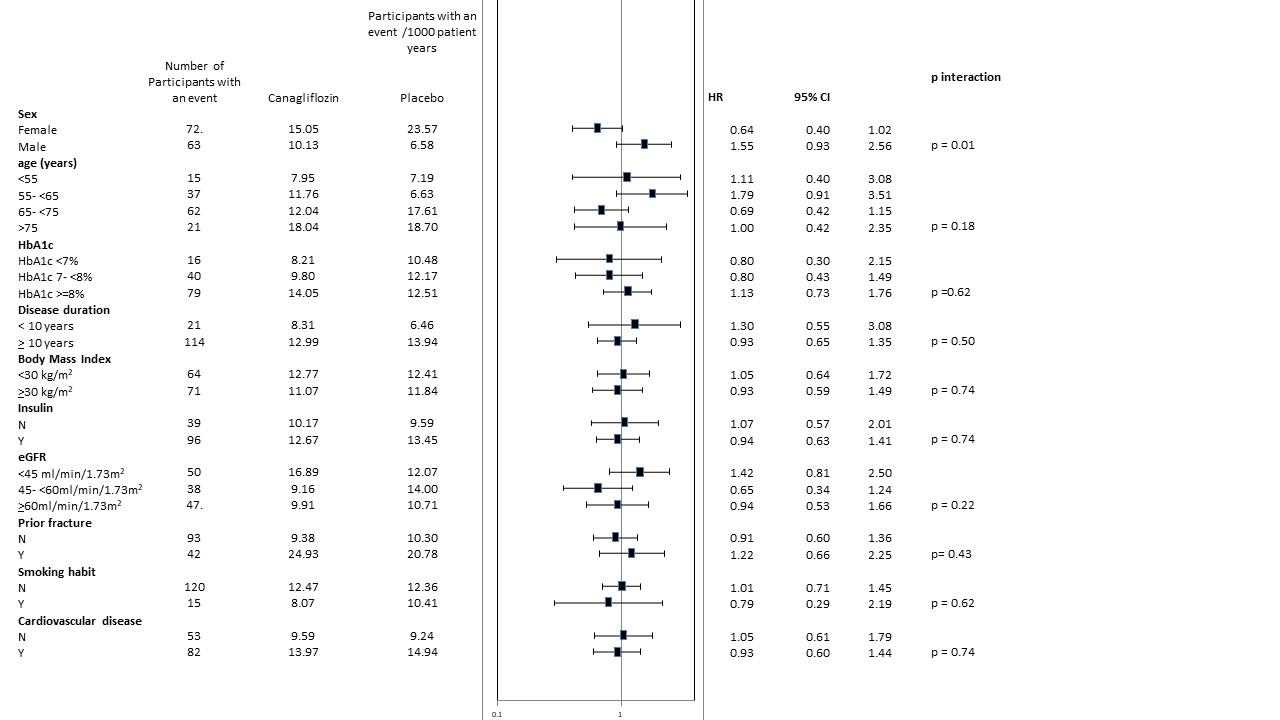

Supplement: Supplementary 2 — Supplementary Figure 2: fracture events according to canagliflozin versus placebo across baseline subgroups. [file 9998891.f2.jpg]
