# Supplementary material for: Risk Factors for Fracture in Patients with Coexisting Chronic Kidney Disease and Type 2 Diabetes: An Observational Analysis from the CREDENCE Trial
Source: J Diabetes Res. 2022 May 27;2022:9998891. doi: 10.1155/2022/9998891 (PMC9168808; doi:10.1155/2022/9998891)
Supplement: Supplementary 3 — Supplementary Table 1: baseline characteristics of participants. Supplementary Table 2: fractures according to the skeletal site. Supplementary Table 3: univariate Cox-proportional models for fracture. [file 9998891.f3.docx]

## Supplementary Table 1 Baseline Characteristics of Participants

| Characteristic | Canagliflozin (N = 2202) | Placebo (N = 2199) | All Patients (N = 4401) |
| --- | --- | --- | --- |
| Age — yr | 62.9±9.2 | 63.2±9.2 | 63.0±9.2 |
| Female sex — no. (%) | 762 (34.6) | 732 (33.3) | 1494 (33.9) |
| Race or ethnic group — no. (%) | | | |
| White | 1487 (67.5) | 1444 (65.7) | 2931 (66.6) |
| Black | 112 (5.1) | 112 (5.1) | 224 (5.1) |
| Asian Race | 425 (19.3) | 452 (20.6) | 877 (19.9) |
| Other | 178 (8.1) | 191 (8.7) | 369 (8.4) |
| Current smoker — no. (%) | 341 (15.5) | 298 (13.6) | 639 (14.5) |
| Duration of diabetes — years | 15.5±8.7 | 16.0±8.6 | 15.8±8.6 |
| Cardiovascular disease — no. (%) | 1113 (50.5) | 1107 (50.3) | 2220 (50.4) |
| Blood pressure — mm Hg | | | |
| Systolic | 139.8±15.6 | 140.2±15.6 | 140.0±15.6 |
| Diastolic | 78.2±9.4 | 78.4±9.4 | 78.3±9.4 |
| Glycated hemoglobin — % | 8.3±1.3 | 8.3±1.3 | 8.3±1.3 |
| Estimated GFR — ml/min/1.73 m^2 *^ | 56.3±18.2 | 56.0±18.3 | 56.2±18.2 |
| Median urinary albumin-to-creatinine ratio (IQR) | 923 (459–1794) | 931 (473–1868) | 927 (463–1833) |

* Estimated glomerular filtration (eGFR) was calculated using the CKD-EPI (CKD Epidemiology Collaboration) formula

Supplementary Table 2 : Fractures according to the skeletal site

|  | | Canagliflozin | Placebo | Total *(%)* |
| --- | --- | --- | --- | --- |
| Total Number of fractures | | 67 | 68 | 135 *(100)* |
| Upper Limb Fractures | | 18 | 21 | 39 *(28.9)* |
| Lower Limb Fracture | | 32 | 32 | 64 *(47.4)* |
| Spine |  | 3 | 6 | 9 *(6.7)* |
| Other |  | 14 | 9 | 23 *(17.0)* |

## Supplementary Table 3: Univariate Cox-Proportional Models for Fracture:

|  | Hazard Ratio | 95% Confidence Interval | p-value |
| --- | --- | --- | --- |
|  |  |  |  |
| **Traditional Risk Factors** |  |  |  |
| Sex Female | 2.3 | 1.64 – 3.23 | <0.001 |
| Age (Years) | 1.03 | 1.01 – 1.05 | < 0.001 |
| Prior History of Fracture (Y) | 2.31 | 1.60 – 1.33 | <0.001 |
| **General Population Exploratory Factors** |  |  |  |
| Asian Race (ref white) | 1.29 | 0.86 – 1.94 | 0.22 |
| Black (ref white) | 0.44 | 0.14 – 1.39 | 0.16 |
| Other (ref. White) | 1.29 | 0.72 – 2.32 | 0.39 |
| Serum Albumin (g/dL) | 0.49 | 0.32 – 0.74 | <0.001 |
| Thyroid hormone use (Y) | 1.56 | 0.96 – 2.54 | 0.07 |
| Proton Pump Inhibitor use (Y) | 1.46 | 0.99 – 2.14 | 0.06 |
| Calcium supplement use (Y) | 1.79 | 0.79 – 4.06 | 0.17 |
| Vitamin D therapy (Y) | 2.1 | 1.39 – 3.19 | <0.001 |
| Beta Blocker use (Y) | 1 | 0.71 – 1.40 | 0.98 |
| **CKD- MBD factors** |  |  |  |
| Baseline UACR (mg/g) | 1.00 | 1.00 – 1.00 | 0.88 |
| Baseline eGFR (mL/min/1.73m2) | 0.99 | 0.97 – 1.00 | 0.09 |
| Magnesium (mg/dL) | 0.67 | 0.35 – 1.28 | 0.22 |
| Phosphate (mg/dL) | 1.15 | 0.87 – 1.52 | 0.33 |
| Serum calcium (mg/dL) | 0.78 | 0.56 – 1.09 | 0.15 |
| Bicarbonate (mEq/L) | 1.01 | 0.96 – 1.07 | 0.65 |
| Alkaline Phosphatase (mIU/L) | 1.00 | 1.00 – 1.01 | 0.08 |
| Sodium (mEQ/L) | 1.03 | 0.97 – 1.10 | 0.29 |
| Urate (mg/dL) | 1.01 | 0.91 – 1.11 | 0.89 |
| **Diabetes Related Parameters** |  |  |  |
| Diabetes duration (years) | 1.01 | 0.99 – 1.03 | 0.32 |
| Baseline BMI (kg/m2) | 0.98 | 0.96 – 1.01 | 0.24 |
| Baseline Hemoglobin A1c (%) | 1.13 | 1.00 – 1.28 | 0.05 |
| Insulin Use | 1.28 | 0.88 – 1.86 | 0.2 |
| History of retinopathy (Y) | 1.17 | 0.83 – 1.64 | 0.37 |
| History of neuropathy (Y) | 1.21 | 0.86 – 1.70 | 0.27 |
| **Cardiovascular Risk Factors** |  |  |  |
| Statin use (Y) | 1.43 | 0.96 – 2.14 | 0.08 |
| Baseline SBP (mmHg) | 1 | 0.99 – 1.01 | 0.59 |
| Baseline Triglycerides (mmol/L) | 0.98 | 0.88 – 1.09 | 0.7 |
| Baseline LDL-C (mmol/L) | 0.97 | 0.82 – 1.14 | 0.7 |
| Smoking Habit (Y) | 0.75 | 0.44 – 1.29 | 0.31 |
| History of CV Disease (Y) | 1.51 | 1.07 – 2.14 | 0.02 |

**List of Abbreviations contained in Supplementary Table 3:**

Chronic Kidney Disease Mineral Bone Disorder (CKD-MBD)

Urine Albumin-to-Creatinine Ratio (UACR)

Body-mass index (BMI)

Systolic Blood Pressure (SBP)

low-density lipoprotein-cholesterol (LDL-C)

Cardiovascular (CV)

Estimated Glomerular filtration rate (eGFR) (Note : Estimated glomerular filtration was calculated using the CKD-EPI (CKD Epidemiology Collaboration) formula)
